# Research on variable universe fuzzy PID control for semi-active suspension with CDC dampers based on dynamic adjustment functions

**DOI:** 10.1038/s41598-024-54152-3

**Published:** 2024-02-11

**Authors:** Guanggang Ji, Lidong Zhang, Mingda Cai, Xianke Meng, Zhengyu Du, Jiuhong Ruan, Shenhao Guan, Zhiwen Liu

**Affiliations:** 1 School of Rail Transportation, Shandong Jiaotong University, Jinan, 250000 China; 2 State Key Laboratory of Mechanical Behavior and System Safety of Traffic Engineering Structures, Shijiazhuang Tiedao University, Shijiazhuang, 050043 China

**Keywords:** Energy science and technology, Engineering

## Abstract

The vehicle suspension system is a complex system with multiple variables, nonlinearity and time-varying characteristics, and the traditional variable universe fuzzy PID control algorithm has the problems of over-reliance on expert experience and non-adaptive adjustment of the contracting-expanding factor parameters, which make it difficult to achieve a better control effect. In this paper, the system error *e*(*t*) and its change rate *ec*(*t*) are introduced into the contracting-expanding factor as dynamic parameters to realize the adaptive adjustment of the contracting-expanding factor parameters, and propose a variable universe fuzzy PID control based on dynamic adjustment functions (VUFP-DAF), which uses the real-time contracting-expanding factor to realize the adaptive adjustment of the fuzzy universe, so as to improve the ride comfort of vehicles. The research results show that the proposed VUFP-DAF has strong adaptability and can effectively improve the ride comfort and handling stability of vehicles under different speeds and road excitations, providing a certain technical basis for the development of the semi-active suspension system.

## Introduction

As the connecting part between the wheel and the body, the suspension system can effectively improve the ride comfort and handling stability of vehicles. According to the different forms of control force generation, the suspension system is mainly divided into passive suspension, active suspension, semi-active suspension and energy feedback suspension^1^. A large number of scholars at home and abroad have conducted in-depth research on the above four suspensions, and fruitful results have been achieved. Among them, the semi-active suspension can adjust the magnitude of damping force in real time according to the driving conditions, and can achieve similar damping effect as the active suspension, with the advantages of low energy consumption, high reliability and simple structure^2^, which has become the hot issue of vehicle dynamics.

For the design and optimization of semi-active suspension controllers, researchers have proposed the optimal control, sliding mode control, PID control, neural network control, fuzzy control^3–6^. For example, Attia et al.^7^ designed a linear quadratic regulator (LQR) based on the optimal control theory to improve the smoothness of vehicles and maintain the stability of roads, but its robustness is poor. When the working conditions change, the control effect is no longer optimal. In order to improve the dynamic performance of vehicle semi-active suspension system with parameter uncertainty and actuator failure under external road interference, Pang et al.^8^ proposed an improved adaptive sliding mode control strategy. The effectiveness and robustness of the controller were verified through numerical simulation, but the control switching process is prone to jitter and affects the control effect. Yuan et al.^9^ proposed a kind of control strategy that combines PID control with particle swarm optimization algorithm, which can combine the advantages of PID control with the powerful search ability of particle swarm algorithm to optimize the PID controller parameters. The research results show that this control strategy further improves the control effect of PID controller. However, both optimal control and sliding mode control algorithms are based on the establishment of the accurate mathematical model of the control system. For the mathematical model of multi-input and multi-output suspension systems, intelligent algorithms with strong self-adaptability have gradually become research highlights. Fuzzy control algorithms, as a nonlinear control method, can be well matched. For example, for the complex and nonlinear environment in which the suspension system is located, Cao et al.^10^ introduced a fuzzy logic system to approximate the unknown nonlinear function and designed an adaptive fuzzy controller to solve the stability problem of suspension systems with random disturbances during the control design process. The effectiveness of the proposed method was verified through simulation experiments, which to some extent improved the ride comfort of vehicles. Pang et al.^11^ designed a variable universe fuzzy controller for semi-active suspensions based on fuzzy neural network, and used back propagation and particle swarm optimization (BP-PSO) algorithm to optimize the contracting-expanding factor parameters, but the online computational cost of algorithms is high for suspension control systems, which easily leads to severe hysteresis and poor robustness. In order to improve the uncertainty between the model error and the external disturbance, Song et al.^12^ proposed an improved fuzzy sliding mode controller for semi-active suspension systems. Through the research on the vibration control performance of the semi-active suspension system with magnetorheological dampers, the effectiveness of the proposed control strategy was verified. Guo et al.^13^ designed a fuzzy PID controller for semi-active suspension systems and verified the damping effect of the controller through simulation experiments under different operating conditions. However, the conventional fuzzy PID controller has the problems of the fixed universe and excessive reliance on expert experience, which make the fuzzy control imprecise in adjusting PID parameters, the system response becomes slow, difficult to stabilize and did not consider the pitch vibration problem of the control system.

For the shortcomings of the traditional fuzzy PID controller, Li et al.^14^ proposed the idea of variable universe fuzzy control, which can effectively improve the adaptivity and control accuracy of systems by using the contracting-expanding factor to adjust the size of fuzzy universes. At present, there is no uniform form for the design of contracting-expanding factors, mainly including functional and fuzzy types. Wang et al.^15^ proposed the variable universe fuzzy control strategy for the vehicle semi-active air suspension, and introduced the functional contracting-expanding factor to change the universe, which to some extent overcomes the defect that the control parameters of conventional controllers cannot be changed once determined, thereby improving control accuracy. In order to improve the ride comfort of vehicles and suppress the coupled vibration of the powertrain mounting system, Chen et al.^16^ proposed a variable universe adaptive fuzzy PID control algorithm, which uses a functional expansion factor to adjust the fuzzy universe of the controller. The research results show that the designed adaptive universe fuzzy PID controller plays the expected control effect in the mounting system, and can effectively reduce the vibration of the powertrain during driving and improve the comfort of vehicles. However, it is difficult to determine the parameters of the functional contracting-expanding factor and accurately describe the real-time change of the contracting-expanding factor during the control process with fixed parameters. To solve this problem, Zhang et al.^17^ designed a variable universe fuzzy controller based on fuzzy reasoning, used the fuzzy controller to replace the traditional function formula, which achieved good control results through simulation research. However, the determination of fuzzy rules of the controller overly relies on expert experience, which has the same problems as traditional fuzzy controllers, making it difficult to ensure control accuracy, and the tedious integration operation make this method unsuitable for practical engineering structures. And both functional and fuzzy scaling factors have certain limitations. In order to achieve adaptive adjustment of the scaling factor parameters, Liu et al.^18^ proposed an improved variable universe fuzzy PID control algorithm on the basis of fuzzy PID control. The fuzzy controller was used to adjust the scaling factor parameters, and the obtained parameters were substituted into the function model to complete the design of the scaling factor controller. Numerical analysis and experimental verification were conducted on a three-layer frame structure with magnetorheological dampers. The research results showed that the vibration amplitudes of floor displacement, velocity, and acceleration controlled by the proposed method are all smaller than the traditional fuzzy PID control. However, due to the nonlinearity and time delay of the control system in the actual control process, in order to achieve better control results, it is often necessary for the fuzzy controller to have complete control rules. Factors such as nonlinearity, time delay, and random interference often affect the judgment of experts and operators, and it is not possible to establish complete fuzzy rules for any control system, thereby affecting the control accuracy of the system. Therefore, how to achieve real-time dynamic design of scaling factor parameters based on system feedback information will be an important research topic.

In view of the above analysis, the scaling factor parameters in variable universe fuzzy control cannot be dynamically designed based on system feedback information. Motivated by improving the performance of suspension systems and realize the real-time dynamic design of scaling factor parameters, the VUFP-DAF control strategy of semi-active suspensions is studied. The main contributions of this paper are summarized as follows:

1. Designing a novel scaling factor controller based on dynamic adjustment functions, which can adjust the scaling factor parameter adaptively according to the real-time feedback information of systems, is more flexible and practical in real applications.
2. Based on stability theory, the rationality of the proposed scaling factor controller was verified, which solves the problem of poor control effect due to the fixed parameters of the functional scaling factor and the limited fuzzy rules of fuzzy scaling factor.
3. Combining the designed scaling factor controller with fuzzy PID controller, the VUFP-DAF control strategy for vehicle semi-active suspension is proposed. The simulation results under different working conditions show that the proposed VUFP-DAF control strategy has strong practicability and good control performance, which provides a new control algorithm for the vibration control of vehicle semi-active suspensions.

## Model of the CDC damper and semi-active suspension

### CDC damper polynomial model

**Figure 1:**
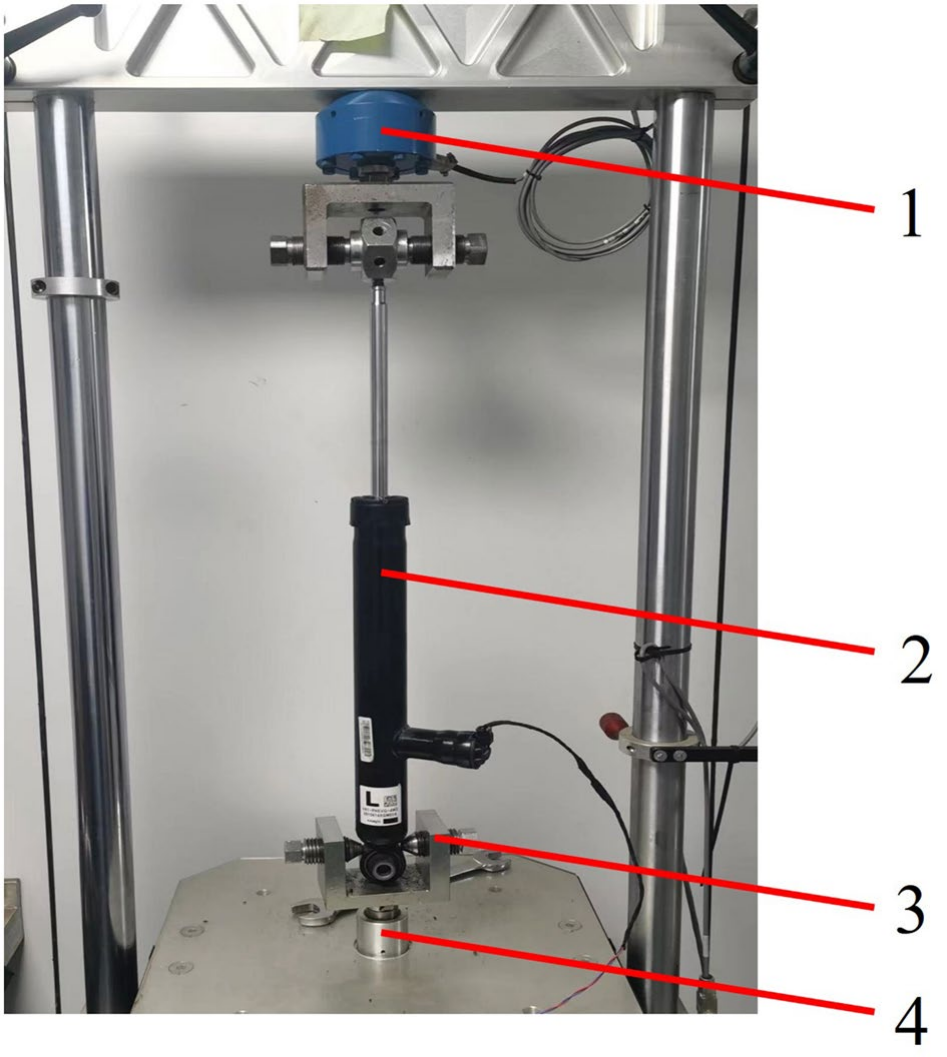
Testing setup of CDC damper. 1. Force sensor. 2. CDC damper. 3. Fixing device. 4. Displacement sensor.

Continuous damping control (CDC) is widely used as a nonlinear element in semi-active suspension systems^19^, but due to its nonlinearity and many other factors, the mechanical model of this damper is difficult to describe accurately. Xia et al.^20^ established the hydraulic and mathematical model of CDC dampers, and verified the validity through simulation analysis, which laid the foundation for the development of the semi-active suspension control strategy. However, most parameters of the model need to be optimally identified and are difficult to solve, which will increase the system delay and reduce the stability in practical applications. Yan et al.^21^ established an analytical model of CDC dampers and verified its effectiveness through bench experiments. Choi et al.^22^ established a non-parametric model of the magnetorheological damper based on polynomial to solve the problem of parametric models, and the simulation results of the non-parametric model is in good agreement with the experimental results and can meet the control requirements of semi-active suspensions. Based on the mechanical characteristic data of the CDC damper measured in the bench test, a polynomial model of the CDC damper was established in this paper, and its external characteristics experiment were shown in Fig. 1. The polynomial model divides the velocity characteristic curve of the damper into a positive acceleration curve and a negative acceleration curve. And the velocity values of the piston rod are used to fit the recovery and compression curves respectively, and the polynomial coefficients are obtained. Then the polynomial model is obtained by linear fitting with the current value. The expressions of the damper damping force and the coefficients in the polynomial are shown in Eqs. (1) and (2).

1 $$ F_{{\text{d}}} = \sum\limits_{i = 0}^{n} {a_{i} v^{i} } $$

2 $$ a_{i} = b_{i} I + c_{i} $$

where *a*_*i*_ is the fitting polynomial coefficient obtained from the experimental results, the coefficients *b*_*i*_ and *c*_*i*_ can be obtained from the linear data, *v* is the relative speed of the damper piston rod, *I* is the input current of dampers, and *n* is the order of the polynomial. In this paper, the 5th degree polynomial is selected to model the mechanical characteristic of CDC dampers.

**Figure 2:**
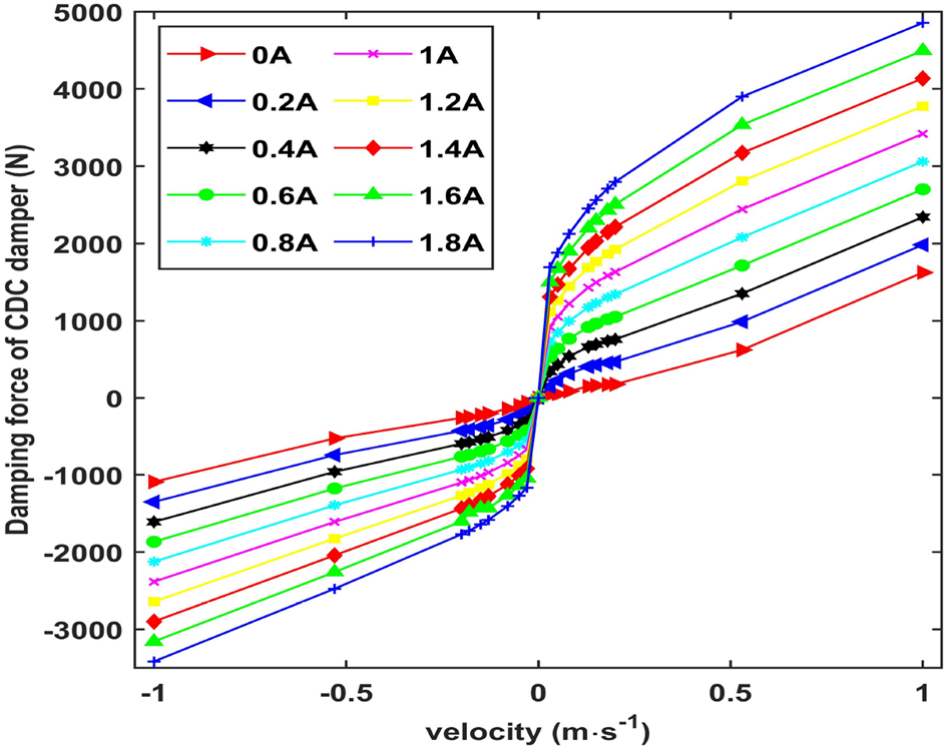
Force–velocity characteristic curves of the CDC damper.

The parameter identification results of the CDC damper were obtained by regression analysis, as shown in Table 1. The CDC damper polynomial model is obtained by substituting the relevant parameters into Eq. (1). According to the CDC damper model, the damping force–velocity characteristic curves of the CDC damper are obtained, as shown in Fig. 2.

**Table 1:** Parameter identification results.

*i*	Stretch model parameters(*v* > 0)		Compression model parameters(*v* < 0)	
	*b*_*i*_	*c*_*i*_	*b*_*i*_	*c*_*i*_
0	857.9	− 174.3	− 563.2	25.54
1	3670	5119	2032	2910
2	− 2932	− 27,600	4589	11,280
3	− 3584	64,890	7572	23,490
4	5835	− 57,290	5889	19,680
5	− 2053	16,880	1601	5679

### Semi-active suspension system dynamics model

**Figure 3:**
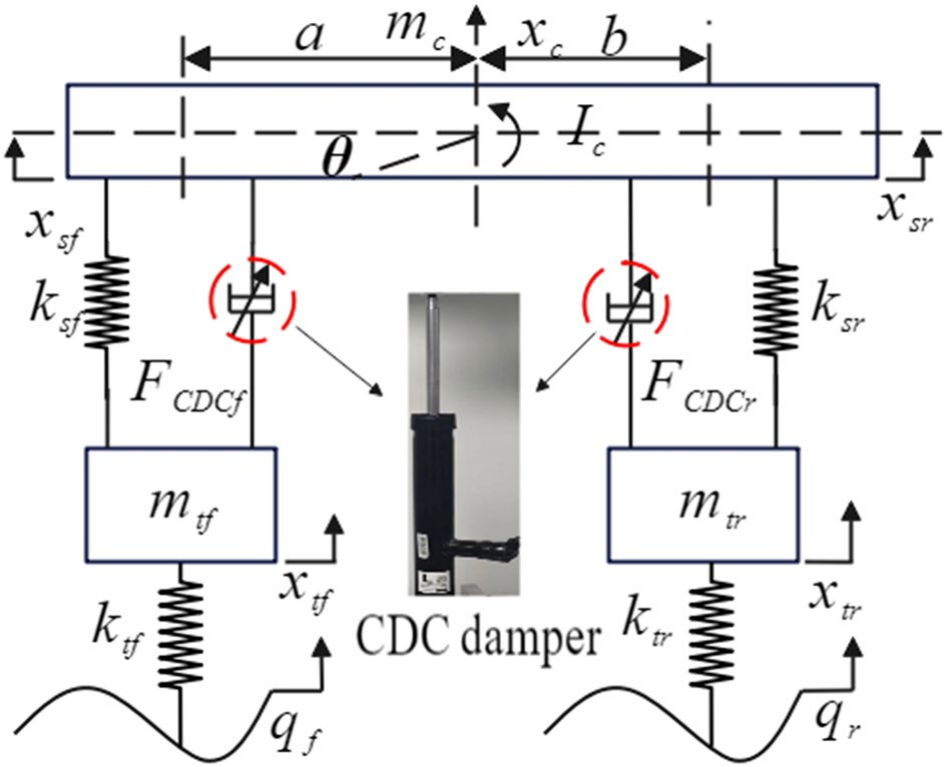
1/2 vehicle semi-active suspension system.

The 1/2 vehicle suspension system is simple in structure, contains the main features of vehicle dynamics analysis, and is widely used in the study of suspension systems. Figure 3 shows the structure of 1/2 vehicle semi-active suspension systems.

Where *m*_*c*_ is the sprung mass, *m*_*tf*_ and *m*_*tr*_ are the unsprung masses of the front and rear suspensions respectively, *I*_*c*_ is the moment of inertia, *x*_*c*_ and *θ* are the vertical and pitch angular displacements at the gravity center of vehicle body respectively, *x*_*tf*_ and *x*_*tr*_ are the vertical displacements of the front and rear unsprung masses respectively, *q*_*f*_ and *q*_*r*_ are the road excitations at the front and rear wheels respectively, *k*_*sf*_ and *k*_*sr*_ are the stiffness coefficients of the front and rear suspensions respectively, *k*_*tf*_ and *k*_*tr*_ are the stiffness coefficients of the front and rear tires respectively, *F*_CDC*f*_ and *F*_CDC*r*_ are the damping forces of the front and rear suspensions respectively; *a* and *b* are the distances from the front and rear axles to its center.

Assuming that the left and right sides of 1/2 vehicle suspension system are completely symmetrical, and considering the force balance between the vertical and pitch directions of the vehicle body and the vertical direction of wheels, this paper carried out the dynamic analysis of 1/2 vehicle suspension systems, and established the dynamic equation of 1/2 vehicle suspension systems as shown in Eq. (3).

3 $$ \left\{ \begin{gathered} m_{c} \ddot{x}_{c} + k_{sf} (x_{sf} - x_{tf} ) + k_{sr} (x_{sr} - x_{tr} ) + F_{CDCf} + F_{CDCr} = 0 \hfill \\ I_{c} \ddot{\theta }_{c} - ak_{sf} (x_{sf} - x_{tf} ) + bk_{sr} (x_{sr} - x_{tr} ) - aF_{CDCf} + bF_{CDCr} = 0 \hfill \\ m_{tf} \ddot{x}_{tf} - k_{sf} (x_{sf} - x_{tf} ) + k_{tf} (x_{tf} - q_{f} ) - F_{CDCf} = 0 \hfill \\ m_{tr} \ddot{x}_{tr} - k_{sr} (x_{sr} - x_{tr} ) + k_{tr} (x_{tr} - q_{r} ) - F_{CDCr} = 0 \hfill \\ x_{sf} = x_{c} - a\theta \hfill \\ x_{sr} = x_{c} + b\theta \hfill \\ \end{gathered} \right. $$

Take ${\mathbf{X}} = [x_{sf} ,x_{tf} ,x_{sr} ,x_{tr} ,\dot{x}_{sf} ,\dot{x}_{tf} ,\dot{x}_{sr} ,\dot{x}_{tr} ]$,${\mathbf{U}} = [F_{CDCf} ,F_{CDCr} ,q_{{_{f} }} ,q_{{_{r} }} ]$ and ${\mathbf{Y}} = [x_{sf} - x_{tf} ,k_{tf} (x_{tf} - q_{f} ),x_{sr} - x_{tr} ,k_{tr} (x_{tr} - q_{r} ),\ddot{m}_{c} ,\ddot{\theta }_{c} ,\dot{x}_{sf} - \dot{x}_{tf} ,\dot{x}_{sr} - \dot{x}_{tr} ]$ as the system state variables, the input and output variables of the suspension system, respectively. The system state space equation can be obtained from Eq. (3).

4 $$ \left\{ \begin{gathered} {\mathbf{\dot{X} = AX + BU}} \hfill \\ {\mathbf{Y = CX + DU}} \hfill \\ \end{gathered} \right. $$

Define $${\mathbf{A = }}\left[ {\begin{array}{*{20}c} {{\mathbf{0}}_{{{\mathbf{4}} \times {\mathbf{4}}}} } & {{\mathbf{E}}_{{{\mathbf{4}} \times {\mathbf{4}}}} } \\ {{\mathbf{A}}_{{{\mathbf{21}}}} } & {{\mathbf{0}}_{{{\mathbf{4}} \times {\mathbf{4}}}} } \\ \end{array} } \right]$$, $$k_{1} = I_{c} + a^{2} m_{c} ,k_{2} = I_{c} + b^{2} m_{c} ,k_{3} = I_{c} - abm_{c} ,k_{4} = I_{c} m_{c}$$, then.

$$ {\mathbf{A}}_{{{\mathbf{21}}}} = \left[ {\begin{array}{*{20}c} { - \frac{{k_{1} }}{{k_{4} }}k_{sf} } & {\frac{{k_{1} }}{{k_{4} }}k_{sf} } & { - \frac{{k_{3} }}{{k_{4} }}k_{sr} } & {\frac{{k_{3} }}{{k_{4} }}k_{sr} } \\ {\frac{{k_{sf} }}{{m_{tf} }}} & { - \frac{{k_{sf} + k_{tf} }}{{m_{tf} }}} & 0 & 0 \\ { - \frac{{k_{3} }}{{k_{4} }}k_{sf} } & {\frac{{k_{3} }}{{k_{4} }}k_{sf} } & { - \frac{{k_{2} }}{{k_{4} }}k_{sr} } & {\frac{{k_{2} }}{{k_{4} }}k_{sr} } \\ 0 & 0 & {\frac{{k_{sr} }}{{m_{tr} }}} & { - \frac{{k_{sr} + k_{tr} }}{{m_{tr} }}} \\ \end{array} } \right],{\mathbf{B}} = \left[ {\begin{array}{*{20}c} 0 & 0 & 0 & 0 & { - \frac{{k_{1} }}{{k_{4} }}} & {\frac{1}{{m_{tf} }}} & { - \frac{{k_{3} }}{{k_{4} }}} & 0 \\ 0 & 0 & 0 & 0 & { - \frac{{k_{3} }}{{k_{4} }}} & 0 & { - \frac{{k_{2} }}{{k_{4} }}} & {\frac{1}{{m_{tr} }}} \\ 0 & 0 & 0 & 0 & 0 & {\frac{{k_{tf} }}{{m_{tf} }}} & 0 & 0 \\ 0 & 0 & 0 & 0 & 0 & 0 & 0 & {\frac{{k_{tr} }}{{m_{tr} }}} \\ \end{array} } \right]^{T} . $$

$$ {\mathbf{C}} = \left[ {\begin{array}{*{20}c} 1 & { - 1} & 0 & 0 & 0 & 0 & 0 & 0 \\ 0 & {k_{tf} } & 0 & 0 & 0 & 0 & 0 & 0 \\ 0 & 0 & 1 & { - 1} & 0 & 0 & 0 & 0 \\ 0 & 0 & 0 & {k_{tr} } & 0 & 0 & 0 & 0 \\ { - \frac{{k_{sf} }}{{m_{c} }}} & {\frac{{k_{sf} }}{{m_{c} }}} & { - \frac{{k_{sr} }}{{m_{c} }}} & {\frac{{k_{sr} }}{{m_{c} }}} & 0 & 0 & 0 & 0 \\ {\frac{a}{{I_{c} }}k_{sf} } & { - \frac{a}{{I_{c} }}k_{sf} } & { - \frac{b}{{I_{c} }}k_{sr} } & {\frac{b}{{I_{c} }}k_{sr} } & 0 & 0 & 0 & 0 \\ 0 & 0 & 0 & 0 & 1 & { - 1} & 0 & 0 \\ 0 & 0 & 0 & 0 & 0 & 0 & 1 & { - 1} \\ \end{array} } \right],{\mathbf{D}} = \left[ {\begin{array}{*{20}c} 0 & 0 & 0 & 0 \\ 0 & 0 & { - k_{tf} } & 0 \\ 0 & 0 & 0 & 0 \\ 0 & 0 & 0 & { - k_{tr} } \\ { - \frac{1}{{m_{c} }}} & { - \frac{1}{{m_{c} }}} & 0 & 0 \\ {\frac{a}{{I_{c} }}} & { - \frac{b}{{I_{c} }}} & 0 & 0 \\ 0 & 0 & 0 & 0 \\ 0 & 0 & 0 & 0 \\ \end{array} } \right]. $$

## The controller of VUFP-DAF

### Traditional variable universe fuzzy PID controller

**Figure 4:**
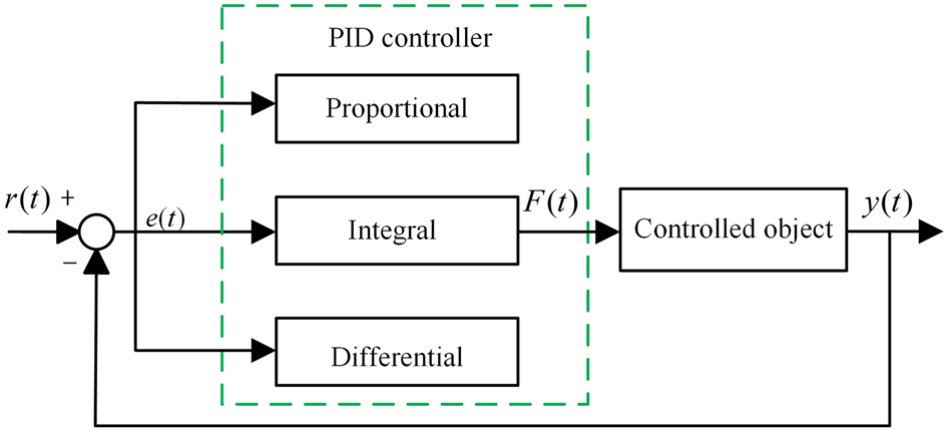
The structure of PID controller.

Let the target response be *r*(*t*) and the actual response be *y*(*t*) of the control system, and the error between them be *e*(*t*), namely, *e*(*t*) = *r*(*t*)-*y*(*t*). The PID algorithm obtains the control force *F*(*t*) required for the vibration control of suspension systems by a linear combination of proportional, integral and differential of *e*(*t*)^23^. The structure of PID controller is shown in Fig. 4.

5 $$ F(t) = K_{p} e(t) + K_{i} \int_{0}^{t} {e(t)dt + K_{d} } \frac{de\left( t \right)}{{dt}} $$

where, *K*_*p*_, *K*_*i*_, *K*_*d*_ are proportional, integral and differential coefficients, respectively. The proportional (P) controller can be used to proportionally reflect the error signal of the control system, when the error occurs, it will immediately generate control force to reduce the error, but not eliminate the error. The integral (I) controller can be used to eliminate steady-state errors and achieve the desired output of the system in steady-state. However, the integral control may cause slow system response, and even overshoot and oscillation. The differential (D) controller has the characteristics of overshooting and prediction, and can predict the trend of the error, which can offset the influence of the delay factor. That is, appropriate differential control can reduce the overshoot of the system and increase its stability. In some applications, only one or two controllers may be enough for the desired system control. Such controllers as.

P, PD and PI may be preferred in some situations^24,25^. In response to the issues of frequent transitions between operating conditions in pumped storage units and the limited adaptability of conventional control to varying operating conditions, Feng et al. proposed the innovative adaptive fuzzy PI controller (IAFPI), incorporating fuzzy control theory, with water head and power as fuzzy inference input variables. The results show that the designed controller has good adaptivity and provides a new approach to the adaptive control problem of pumped storage units^26^. Deb et al. investigated the viability of three different continuous (P, PI, and PID) controllers to meet specific thermal requirements at a desired location in a cooling system with discrete heat sources. The research results indicate that implementing a PID controller action exhibits similarities to the PI controller action. However, lower derivative gains have been observed to enhance oscillation control and reduce response time. An optimal system response can be achieved by appropriately tuning the gains, facilitating improved thermal system management^27^. When the dynamic response of the control system is not required to be high, only need to better steady state performance, PI controller can achieve better control results. Vehicle suspension as a time-varying complex system, which requires high dynamic response in the system control process^28^. A PID controller is, therefore, introduced to get more optimum performance, and how to adjust the values of *K*_*p*_, *K*_*i*_, *K*_*d*_ to achieve the optimal combination becomes the key to ensure that the PID control algorithm has better performance.

**Figure 5:**
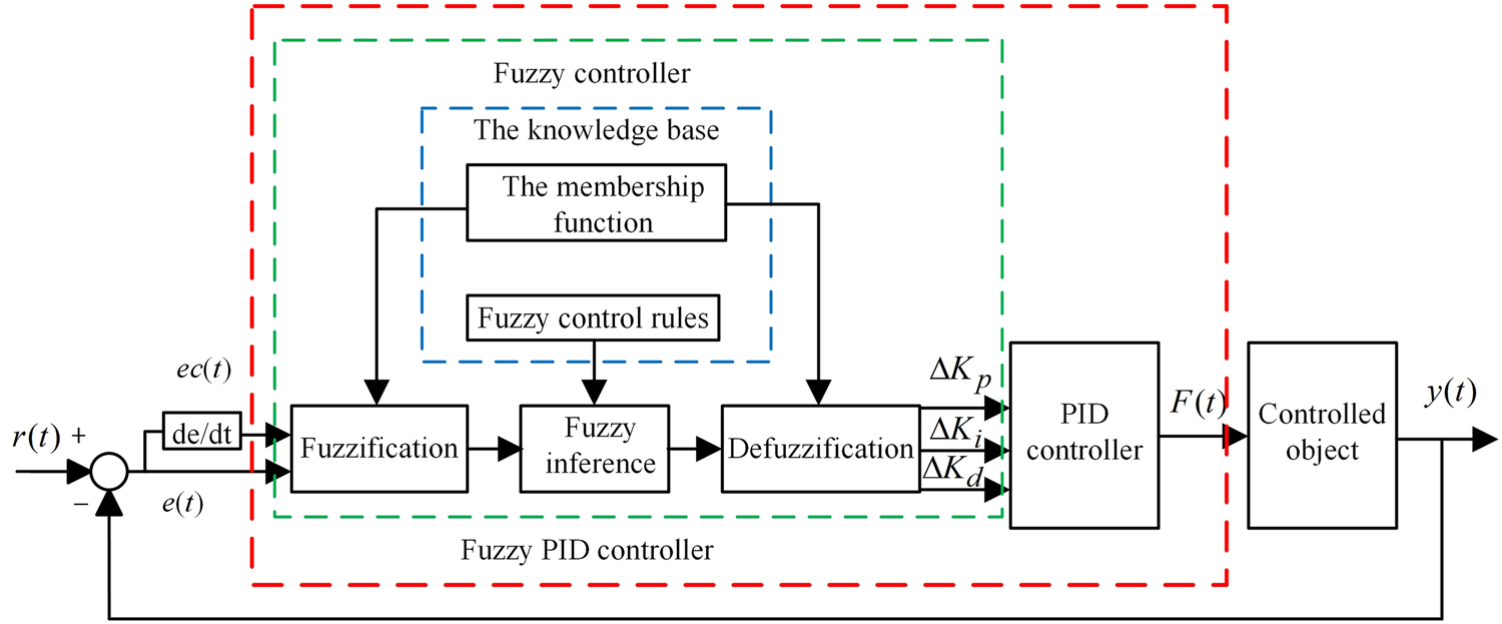
The structure of fuzzy PID controller.

A large number of calculation examples and experiments show that it is difficult to achieve good results using traditional PID controllers with fixed *K*_*p*_, *K*_*i*_ and *K*_*d*_ coefficients^29^. For vehicle suspension system with strong nonlinearity and time-varying characteristics, it is necessary to introduce fuzzy control into PID controller to constitute a fuzzy PID controller to adjust the *K*_*p*_, *K*_*i*_ and *K*_*d*_ coefficients dynamically in real time. Construct a two-input, three-output fuzzy controller, where the inputs of the fuzzy controller are *e*(*t*) and *ec*(*t*), and the outputs are $\Delta K_{p} ,\Delta K_{i} ,\Delta K_{d}$ of the PID controller. Let the initial domains of the input and output variables be *X*_*i*_ = [− *E*_*i*_, *E*_*i*_], *Y*_*j*_ = [− *Q*_*j*_, *Q*_*j*_], respectively, where *E*_*i*_ and *Q*_*j*_ are the domain boundaries. Seven fuzzy subsets of input and output variables are used, namely, NB (negative big), NM (negative medium), NS (negative small), ZE (zero), PS (positive small), PM (positive medium), and PB (positive big), and the affiliation functions are all Gaussian to ensure smooth transition of the algorithm. Then, the size of the output variables $\Delta K_{p} ,\Delta K_{i} ,\Delta K_{d}$ are obtained through three processes: the input fuzzification, the fuzzy inference, and the defuzzification, and thus the proportional, integral, and differential coefficients are adjusted online as shown in Eq. (6). The structure of fuzzy PID controller is shown in Fig. 5. The design process of fuzzy rules for fuzzy PID controllers can be found in reference^30^.

6 $$ F(t) = (K_{p} + \Delta K_{p} )e(t) + (K_{i} + \Delta K_{i} )\int_{0}^{t} {e(t)dt + (K_{d} + \Delta K_{d} )} \frac{de\left( t \right)}{{dt}} $$

**Figure 6:**
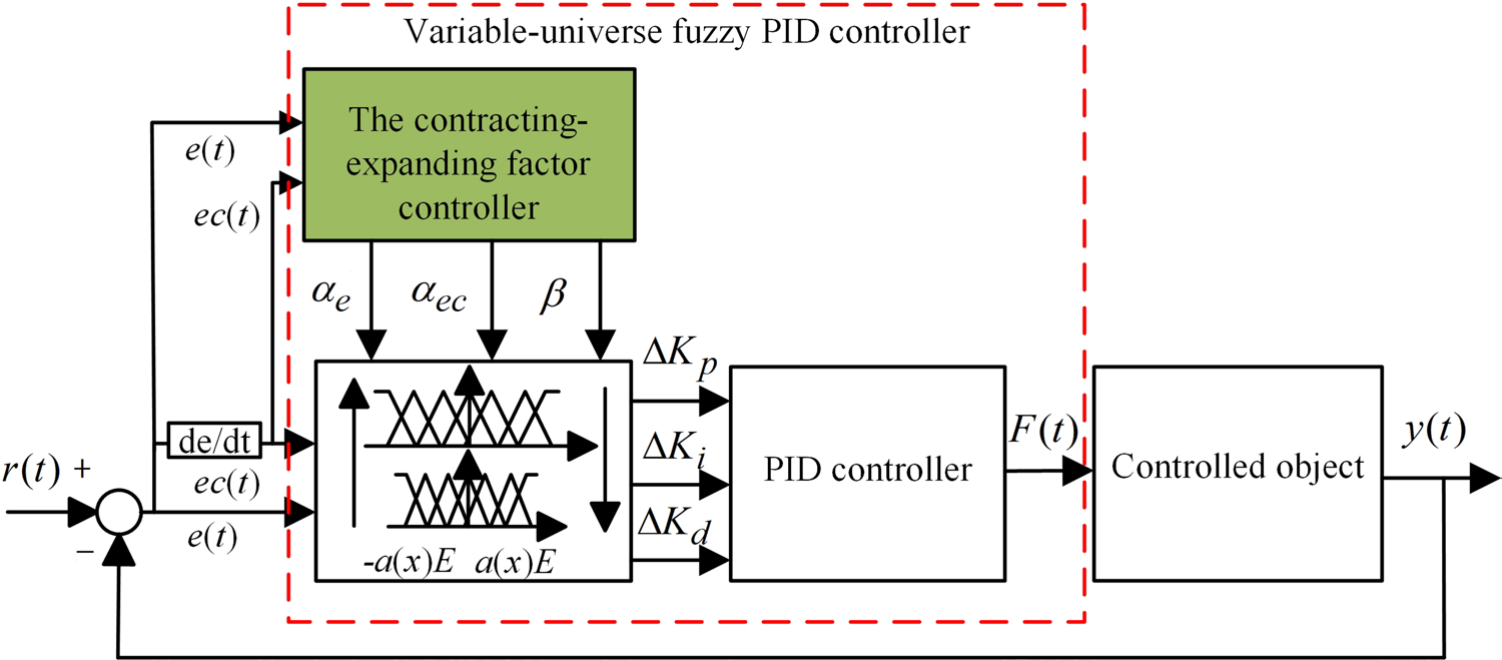
The structure of the variable-universe fuzzy PID controller.

However, the fixed fuzzy domain in the fuzzy PID controller can directly affect the control effect. To address this problem, Cui et al.^31^ proposed a variable-universe fuzzy PID algorithm to improve the control accuracy by using online adjustment of the contracting-expanding factor. The variable-universe fuzzy PID controller is composed of a contracting-expanding factor controller and a fuzzy PID controller. The structure of the variable-universe fuzzy PID controller is shown in Fig. 6. The main function of a contracting-expanding factor controller is to adjust the initial range of the fuzzy universe based on changes in its input variables. When the fuzzy domain shrinks, the membership function will become sharper, resulting in a significant increase in the number of fuzzy control rules near the zero point, thereby improving the accuracy of the control system. When the fuzzy domain expands, the membership function will become wider, and the fuzzy control rules will also be applicable to a larger range, improving the applicability of the fuzzy control rules. That is, the input and output variable scale factors *α*(*e*(*t*)), *α*(*ec*(*t*)) and *β*(*e*(*t*), *ec*(*t*)) are calculated based on *e*(*t*) and *ec*(*t*), and the initial domains of the input and output variables are adjusted using the calculated scale factors *α*(*e*(*t*)), *α*(*ec*(*t*)) and *β*(*e*(*t*), *ec*(*t*)), then the adjusted new domain is shown in Eqs. (7) and (8).

7 $$ X_{i} (x_{i} ) = [ - \alpha_{i} (x_{i} )E_{i} ,\alpha_{i} (x_{i} )E_{i} ] $$

8 $$ Y_{j} (y) = [ - \beta_{j} (y)Q_{j} ,\beta_{j} (y)Q_{j} ] $$

Li et al. design the fuzzy scaling factor controller and propose a variable universe fuzzy control method based on multi-work conditions for flying-walking power line inspection robots, which significantly improves the stability of systems^32^. However, for high-order nonlinear control systems, it is difficult to obtain fuzzy control rules for the contracting-expanding factor controller and there is a problem of over-reliance on expert experience. The functional contracting-expanding factor is definitely a good choice in the actual application for the simple structure and real-time control practicability. Li et al. designed the function contracting-expanding factor controller and proposed a variable universe fuzzy multi-parameter self-tuning PID control strategy for controlling the trolley’s movement. The results indicate that the proposed control strategy exhibits good adaptive ability and robustness, which further improves the stability and safety of the bridge-type bridge crane operation^33^. Therefore, the variable universe fuzzy PID algorithm based on functional contracting-expanding factor is studied in this paper because it has fast system response and directly uses some special functions to design the contracting-expanding factor, avoiding the problem that the control performance of the fuzzy contracting-expanding factor is degraded due to the lack of perfect fuzzy rules. In engineering applications, the more common functional contracting-expanding factor as shown in Eq. (9)^34^.

9 $$ \left\{ {\begin{array}{*{20}l} {\alpha (e(t)) = \left( {\frac{|e(t)|}{{E_{1} }}} \right)^{{\tau_{1} }} + \varepsilon } \hfill \\ {\alpha (ec(t)) = \left( {\frac{|ec(t)|}{{E_{2} }}} \right)^{{\tau_{2} }} + \varepsilon } \hfill \\ {\beta (e(t),ec(t)) = \frac{{\left[ {\left( {\frac{|e(t)|}{{E_{1} }}} \right)^{{\tau_{3} }} + \left( {\frac{|ec(t)|}{{E_{2} }}} \right)^{{\tau_{4} }} } \right]}}{2} + \varepsilon } \hfill \\ \end{array} } \right. $$

where $\varepsilon$ is a sufficiently small positive number, $\tau_{i} (i = 1,2,3,4)$ are design parameters of the contracting-expanding factors. *E*_1_*、E*_2_ are the fuzzy domain boundaries.

### A novel scaling factor controller

The parameters $\tau_{i}$ for the contracting-expanding factor controller are artificially selected constants based on expert experience or similar systems, so the key to designing a functional expansion factor controller is to select the appropriate parameters $\tau_{i}$ to ensure better control effect. Jin et al. designed the structure of contracting-expanding factors and optimized the parameters $\tau_{i}$ by solving an offline optimization problem using the chaotic particle swarm optimization algorithm^35^. However, the optimized $\tau_{i}$ are still fixed constants and cannot be adjusted in real-time based on the dynamic feedback information of the system. The parameters of each vehicle suspension system are different, and the fixed $\tau_{i}$ of contracting-expanding factor cannot achieve the adaptivity of suspension systems. Therefore, it is necessary to design the contracting-expanding factor parameters that can be adjusted in real-time based on the dynamic feedback information of vehicle suspension systems.

This paper designs the scaling factor parameters $\tau_{i}$ that can be adjusted in real time based on *e*(*t*) and *ec*(*t*) in Eq. (10).

10 $$ \tau_{i} = \frac{{E_{1} }}{{|e(t)|(E_{1} + E_{2} ) + \varepsilon }} + \frac{{E_{2} }}{{|ec(t)|(E_{1} + E_{2} ) + \varepsilon }} $$

In order to take the coordination, response speed, steady-state accuracy, overshoot and other performance of the control system into account^36^, when $\tau_{1} > 1$, let $\tau_{1} = 1$, and $\tau_{2} = \tau_{3} = \tau_{4} = \tau_{1}$. Substituting the real-time contracting-expanding factor parameters into Eq. (9), the real-time contracting-expanding factor can be obtained as Eq. (11).

11 $$ \left\{ {\begin{array}{*{20}l} {\alpha (e(t)) = \left( {\frac{|e(t)|}{{E_{1} }}} \right)^{{\tau_{1} }} + \varepsilon } \hfill \\ {\alpha (ec(t)) = \left( {\frac{|ec(t)|}{{E_{2} }}} \right)^{{\tau_{2} }} + \varepsilon } \hfill \\ {\beta (e(t),ec(t)) = \frac{\alpha (e(t)) + \alpha (ec(t))}{2} + \varepsilon } \hfill \\ \end{array} } \right. $$

The contracting-expanding factor should be stable to ensure that the control system can quickly reduce *e*(*t*) and *ec*(*t*). The stability of the real-time contracting-expanding factor is demonstrated in terms of duality, non-zero, monotonicity, regularity, and coordination, respectively^35,37^.

(1) Duality.

When $\alpha (e(t)) = \left( {\frac{|e(t)|}{{E_{1} }}} \right)^{{\frac{{E_{1} }}{{|e(t)|(E_{1} + E_{2} ) + \varepsilon }} + \frac{{E_{2} }}{{|ec(t)|(E_{1} + E_{2} ) + \varepsilon }}}} + \varepsilon$,$\alpha ( - e(t)) = \left( {\frac{| - e(t)|}{{E_{1} }}} \right)^{{\frac{{E_{1} }}{{|e(t)|(E_{1} + E_{2} ) + \varepsilon }} + \frac{{E_{2} }}{{|ec(t)|(E_{1} + E_{2} ) + \varepsilon }}}} + \varepsilon$,$\alpha ( - e(t)) = \alpha (e(t))$.Therefore, *α*(*e*(*t*)) meets the duality property, which ensures that the initial universe always changes in the same proportion during expansion and contraction process. And similarly, *α*(*ec*(*t*)) also meets the duality property.

(2) Non-zero.

When $$e(t) \to 0,\tau_{i} \in [0,1][0,1]$$,$$\mathop {\lim }\limits_{e(t) \to 0} a\left( {e(t)} \right) = \mathop {\lim }\limits_{e(t) \to 0} \left( {\frac{|e(t)|}{{E_{1} }}} \right)^{{\frac{{E_{1} }}{{|e(t)|(E_{1} + E_{2} ) + \varepsilon }} + \frac{{E_{2} }}{{|ec(t)|(E_{1} + E_{2} ) + \varepsilon }}}} + \varepsilon = \varepsilon$$.

Therefore, *α*(*e*(*t*)) meets the non-zero property and ensures that when the value of the input variable is very small, the scaling factor can still regulate the initial universe, avoiding control gaps when the scaling factor is zero. And similarly, *α*(*ec*(*t*)) also meets the non-zero property.

(3) Monotonicity.

When $\alpha (e(t)) = \left( {\frac{|e(t)|}{{E_{1} }}} \right)^{{\frac{{E_{1} }}{{|e(t)|(E_{1} + E_{2} ) + \varepsilon }} + \frac{{E_{2} }}{{|ec(t)|(E_{1} + E_{2} ) + \varepsilon }}}} + \varepsilon$, and *e*(*t*) ∈ [-*E*_1_, *E*_1_], *ec*(*t*) ∈ [-*E*_2_, *E*_2_], then.

$\alpha ^{\prime}(e(t)) = e^{{\left\{ {\frac{{E_{1} }}{{|e(t)|(E_{1} + E_{2} ) + \varepsilon }} + \frac{{E_{2} }}{{|ec(t)|(E_{1} + E_{2} ) + \varepsilon }}} \right\}\ln \left( {\frac{|e(t)|}{{E_{1} }}} \right)}} \left\{ {\left[ {\frac{{E_{1} }}{{|e(t)|(E_{1} + E_{2} )}}} \right]\left[ {1 - \ln \left( {\frac{|e(t)|}{{E_{1} }}} \right)} \right] + \left[ {\frac{{E_{2} }}{{||e(t)|ec(t)|(E_{1} + E_{2} )}}} \right]} \right\} > 0$. *α*(*e*(*t*)) meets the monotonicity property, which ensures the symmetry of the universe and the consistency of contraction ratios. And similarly, *α*(*ec*(*t*)) also meets the monotonicity property.

(4) Regularity.

When *e*(*t*) is the *E*_1_ or -*E*_1_, $\alpha (E_{1} ) = \left( {\frac{{|E_{1} |}}{{E_{1} }}} \right)^{{\frac{{E_{1} }}{{|E_{1} |(E_{1} + E_{2} ) + \varepsilon }} + \frac{{E_{2} }}{{|ec(t)|(E_{1} + E_{2} ) + \varepsilon }}}} + \varepsilon = 1 + \varepsilon \approx 1$.Therefore, *α*(*e*(*t*)) meets the regularity property and ensures that the initial universe boundary (*E*_1_ or -*E*_1_) is meaningful. And similarly, *α*(*ec*(*t*)) also meets the regularity property.

(5) Coordination.

According to Eq. (11), $|e(t)| = E_{1} (\alpha (e(t) - \varepsilon )^{{\frac{1}{{\tau_{1} }}}}$, and $\tau_{1} \le 1$, $\alpha (e(t)) \in [0,1]$, therefore, $|e(t)| \le E_{1} \alpha (e(t)$. *α*(*e*(*t*)) meets the coordination property, which ensures that the adjusted universe does not exceed the initial universe range. And similarly, *α*(*ec*(*t*)) also meets the coordination property.

In Eq. (11), *β*(*e*(*t*), *ec*(*t*)) is a linear combination of *α*(*e*(*t*)) and *α*(*ec*(*t*)), so it is easy to prove that *β*(*e*(*t*), *ec*(*t*)) meets the properties of duality, non-zero, monotonicity, regularity and coordination. Namely, the real-time contracting-expanding factor meets the condition of stability, which makes the variable universe fuzzy control more flexible and suitable for practical applications.

**Figure 7:**
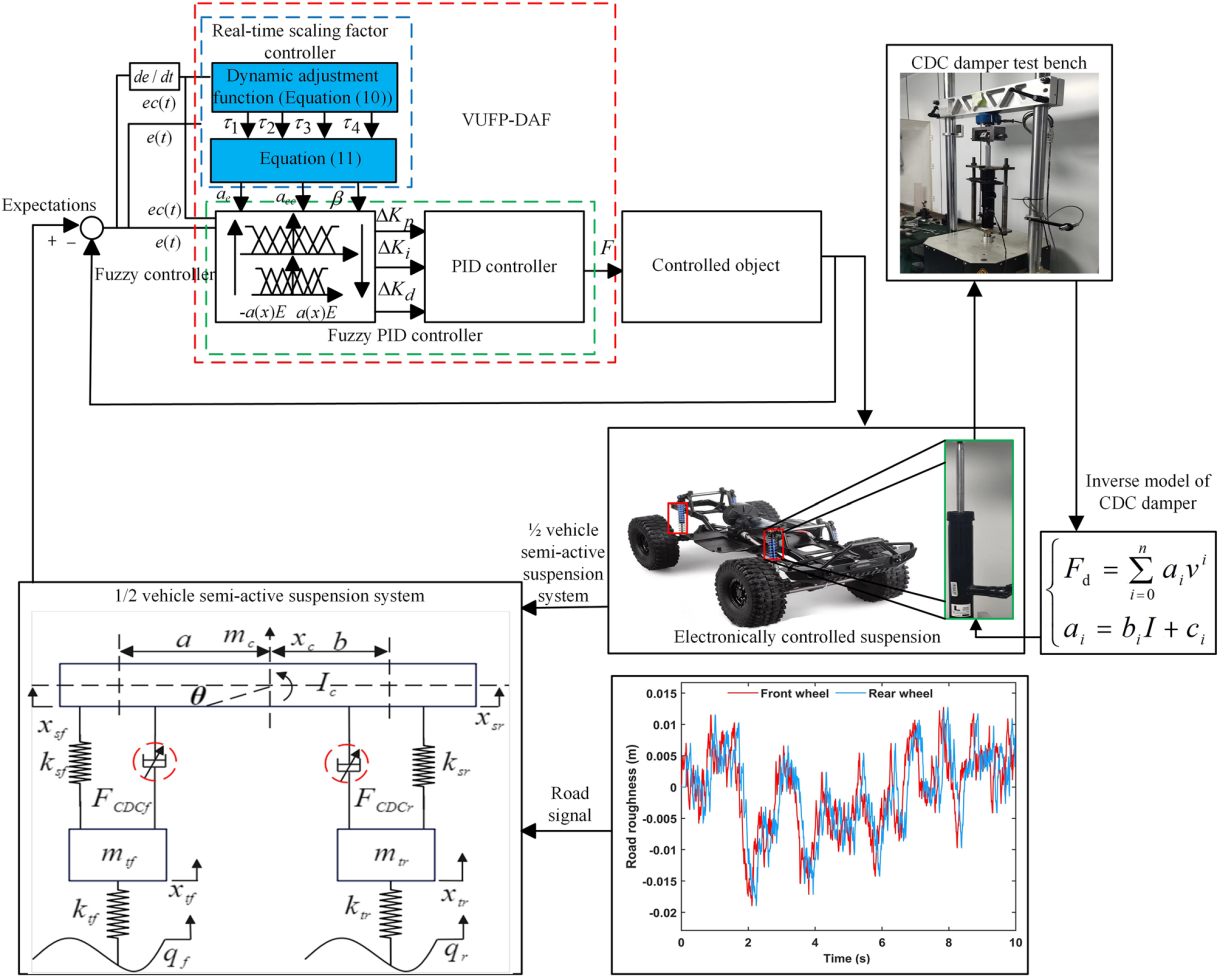
The overall framework of VUFP-DAF control strategy.

And combining the proposed real-time contracting-expanding factor controller with fuzzy PID controller to construct the VUFP-DAF controller. The scaling factor controller obtains real-time input variable scaling factors *α*(*e*(*t*)), *α*(*ec*(*t*)) and output variable scaling factor *β*(*e*(*t*), *ec*(*t*)) based on the system dynamic feedback information *e*(*t*) and *ec*(*t*), and adjusts the fuzzy domain of fuzzy PID controller adaptively by utilizing the obtained scaling factors, so as to achieve the purpose of improving the system control accuracy without changing the number of fuzzy control rules. The overall framework of VUFP-DAF is shown in Fig. 7.

## Simulation results and discussion

According to a domestic SUV vehicle, 1/2 vehicle suspension system parameters are selected as shown in Table 2. The dynamics simulation of semi-active suspension systems with VUFP-DAF is carried out under different conditions, the maximum and root mean square (RMS) values of the vehicle body acceleration (VBA), pitch angle acceleration (PAA), front suspension dynamic deflection (FSD), rear suspension dynamic deflection (RSD), front tire dynamic load (FTDL) and rear tire dynamic load (RTDL) are used as the evaluation indexes of the control effect, and are compared and analyzed with passive suspension (PS), fuzzy PI control (FPI) and variable universe fuzzy PID control based on traditional functional (VUFP-TF) to verify the effectiveness of the proposed VUFP-DAF control strategy in this paper.

**Table 2:** Semi-active suspension system parameters of 1/2 vehicle.

Parameters	Value
*m*_*c*_	974 (kg)
*I*_*c*_	1836.2 (kg/m^2^)
*m*_*tf*_	141 (kg)
*m*_*tr*_	131 (kg)
*k*_*sf*_	39600 (N/m)
*k*_*sr*_	39600 (N/m)
*a*	1.3 (m)
*b*	1.445 (m)
*k*_*tf*_	192000 (N/m)
*k*_*tr*_	192000 (N/m)

### Random road

In this paper, the rational function filtering white noise method^38^ is used to calculate the roughness of random road. The road excitation can be expressed as Eq. (12).

12 $$ \dot{q} = - 0.111\left[ {v_{1} q + 40\sqrt {G_{q} \left( {n_{0} } \right)v} w_{0} (t)} \right] $$

where $G_{q} \left( {n_{0} } \right)$ is the road roughness coefficient,$w_{0} \left( t \right)$ is white noise, $q$ is the road displacement excitation and *v*_1_ is the vehicle speed.

**Figure 8:**
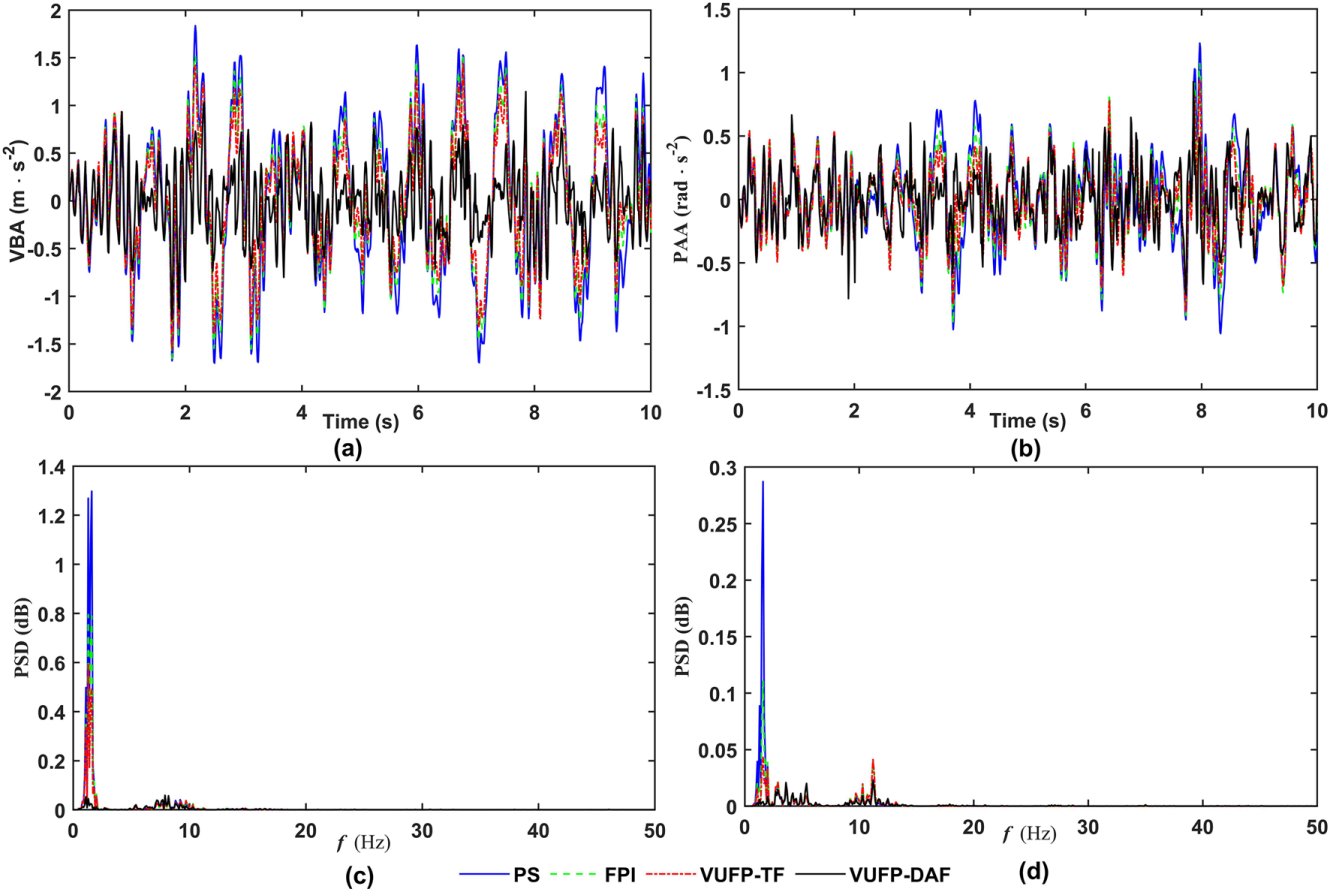
The time-domain response curves of (**a**) VBA, (**b**) PAA and the frequency-domain response curves of (**c**) VBA, (**d**) PAA.

**Figure 9:**
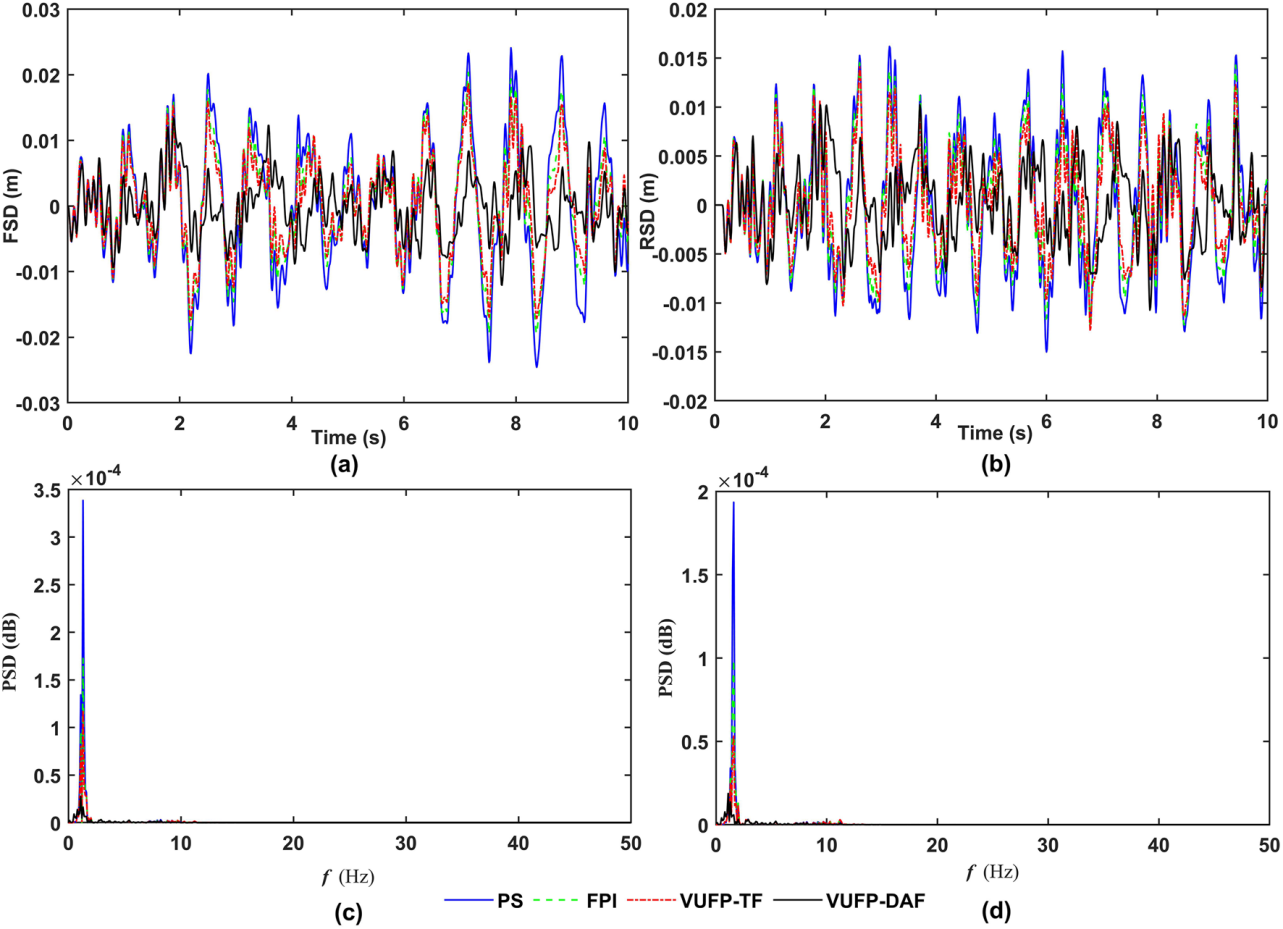
The time-domain response curves of (**a**) FSD, (**b**) RSD and the frequency-domain response curves of (**c**) FSD, (**d**) RSD.

**Figure 10:**
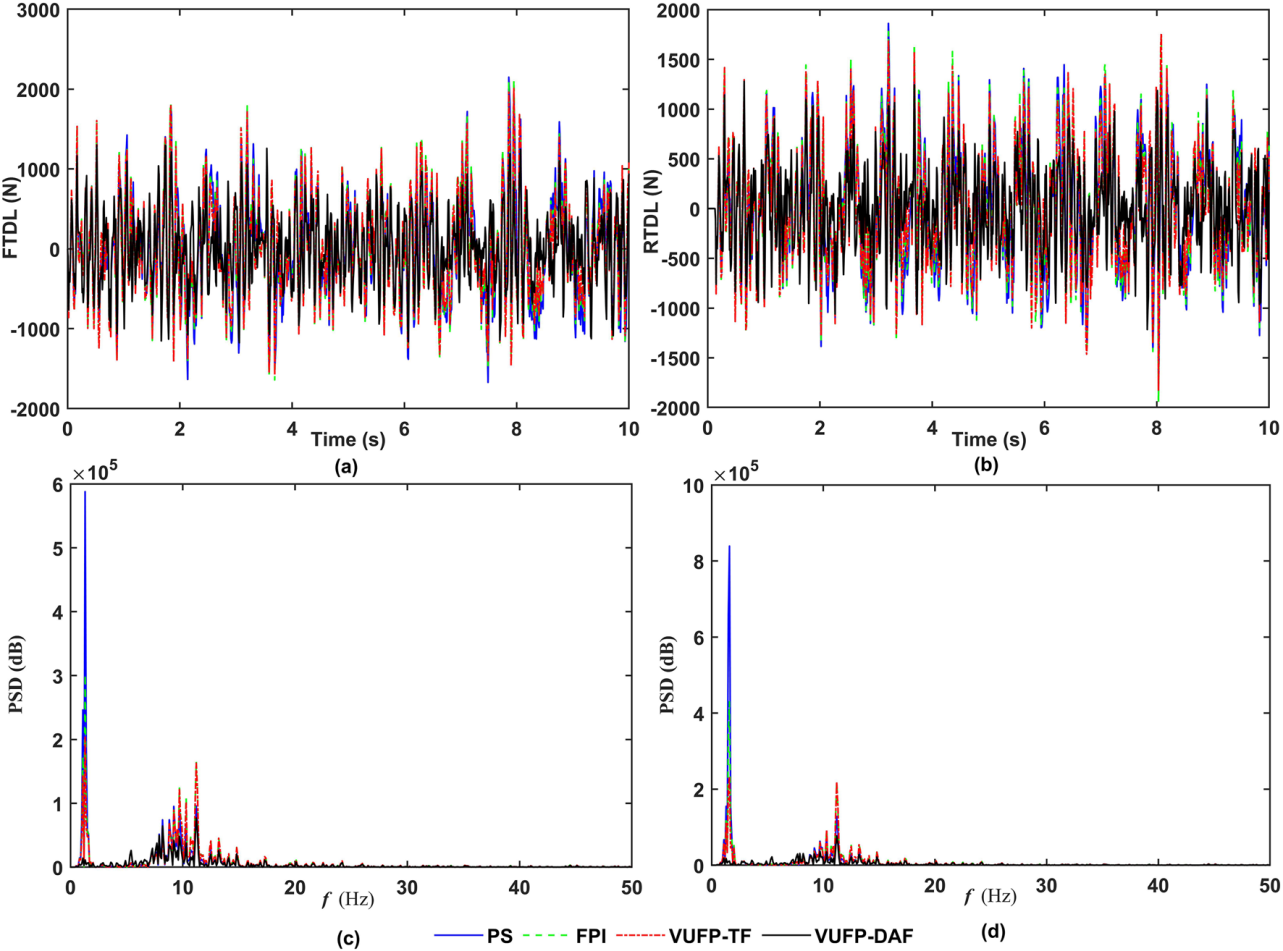
The time-domain response curves of (**a**) FTDL, (**b**) RTDL and the frequency-domain response curves of (**c**) FTDL, (**d**) RTDL.

The time-domain and frequency-domain response curves of VBA, PAA, FSD, RSD, FTDL and RTDL for PS, FPI, VUFP-TF and VUFP-DAF were calculated when the vehicle was driven at 20 m/s on Class B random road as shown in Figs. 8, 9 and 10, respectively.

From Fig. 8, it can be seen that compared to PS, FPI and VUFP-TF, VUFP-DAF controlled VBA and PAA have better control effects. At the same time, the power spectral density (PSD) of VBA and PAA has been improved significantly in the frequency domain response, which greatly improves the ride comfort of vehicles. It can be seen from Figs. 9 and 10 that FSD, RSD, FTDL and RTDL indexes controlled by VUFP-DAF are significantly better than PS, FPI and VUFP-TF, which can effectively reduce the collision probability between the suspension and the limit block, and improve the handling stability of vehicles.

In order to verify the adaptability of VUFP-DAF, the simulation verifications were carried out under the conditions of 10 m/s, 15 m/s, 20 m/s and 25 m/s respectively on Class B and C roads. The RMS values of relevant indicators are shown in Table 3. Under different working conditions, compared with PS, the RMS values of VBA and PAA controlled by VUFP-DAF are reduced by over 50% and 20%, respectively. However, the RMS values of VBA and PAA controlled by FPI and VUFP-TF are reduced by over 10%, 5%, 20% and 10%, respectively. the RMS values of FSD, RSD, FTDL and RTDL controlled by VUFP-DAF are all significantly reduced. The control effect of VUFP-DAF is significantly better than FPI and VUFP-TF, which shows strong adaptability and robustness, and can effectively improve the ride comfort and handling stability of vehicles.

**Table 3:** Performance indexes of suspension systems under different working conditions.

Road (*v*_1_ m/s)	Algorithm	Acceleration		Dynamic deflection		Tire dynamic load	
		Vertical (m/s^2^)	Pitch (rad/s^2^)	Front (m)	Rear (m)	Front (N)	Rear (N)
B(10)	PS	0.5638	0.2428	0.0072	0.0050	451.2	431.9
	FPI	0.4769(↓15.41%)	0.2113(↓12.97%)	0.0057	0.0042	450.4	428.9
	VUFP-TF	0.4246(↓24.69%)	0.1928(↓20.59%)	0.0050	0.0037	434	406
	VUFP-DAF	0.2600(↓53.88%)	0.1693(↓30.27%)	0.0034	0.0029	327.4	301.8
B(20)	PS	0.7803	0.3386	0.0099	0.0069	630.6	604.5
	FPI	0.6626(↓15.08%)	0.2968(↓12.34%)	0.0078	0.0058	630.4	600.4
	VUFP-TF	0.5909(↓24.27%)	0.2713(↓19.88%)	0.0070	0.0051	608.4	570.3
	VUFP-DAF	0.3676(↓41.27%)	0.2368(↓30.06%)	0.0045	0.0039	461.5	424.8
C(10)	PS	1.128	0.4586	0.0144	0.0099	902.4	863.8
	FPI	0.9598(↓14.91%)	0.4247(↓7.39%)	0.0114	0.0084	894.3	852.8
	VUFP-TF	0.8589(↓23.86%)	0.3882(↓15.35%)	0.0102	0.0074	860	807.3
	VUFP-DAF	0.5357(↓52.51%)	0.3497(↓23.75%)	0.0066	0.0056	655.7	599.9
C(20)	PS	1.561	0.6771	0.0198	0.0138	1261	1209
	FPI	1.334(↓14.54%)	0.5976(↓11.74%)	0.0158	0.0117	1252	1193
	VUFP-TF	1.206(↓22.74%)	0.5509(↓18.64%)	0.0142	0.0103	1208	1133
	VUFP-DAF	0.7710(↓50.61%)	0.5011(↓26.23%)	0.0089	0.0076	909.5	842

### Bump road

Bump road is one of the road disturbance forms often used to verify the performance of suspension systems and can be used to simulate deceleration belt or impact excitation. According to the international standard ISO 2361, the mathematical description of bump road can be expressed as Eq. (13).

13 $$ \dot{q} = \left\{ {\begin{array}{*{20}l} {\frac{H}{2}\left( {1 - \cos \left( {\frac{{2\pi v_{1} t}}{L}} \right)} \right)} \hfill & {0 \le t \le \frac{L}{{v_{1} }}} \hfill \\ 0 \hfill & {t > \frac{L}{{v_{1} }}} \hfill \\ \end{array} } \right. $$

where *H* and *L* are the height and length of bump road respectively, taking *H* = 0.005 m, *L* = 5 m^39^. The road excitation of front and rear wheels is the same, only the rear wheel has a certain delay compared to the front wheel, the delay time is $\left( {a + b} \right)/v_{1}$.

**Figure 11:**
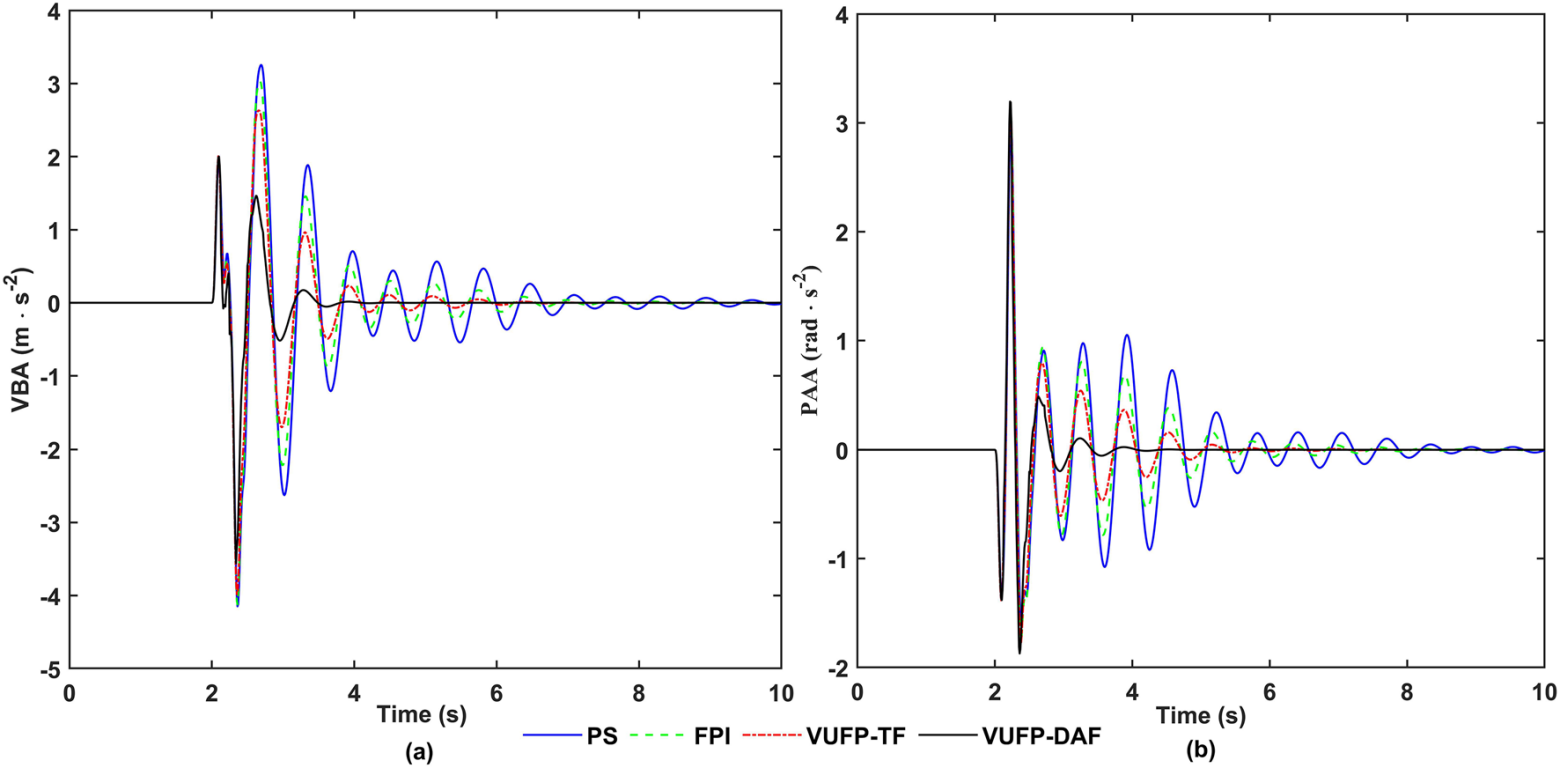
The time-domain response curves of (**a**) VBA, (**b**) PAA.

**Figure 12:**
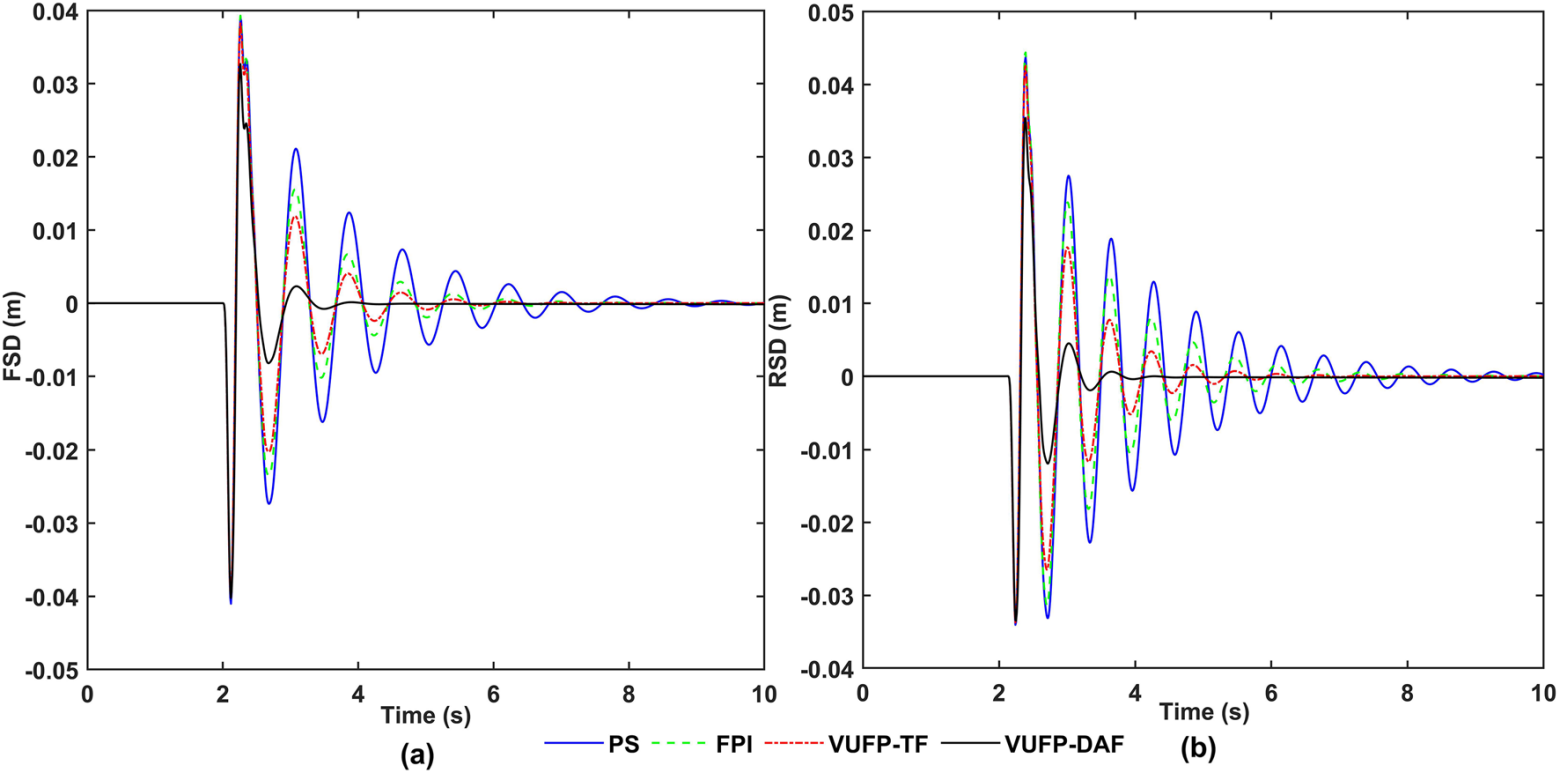
The time-domain response curves of (**a**) FSD, (**b**) RSD.

**Figure 13:**
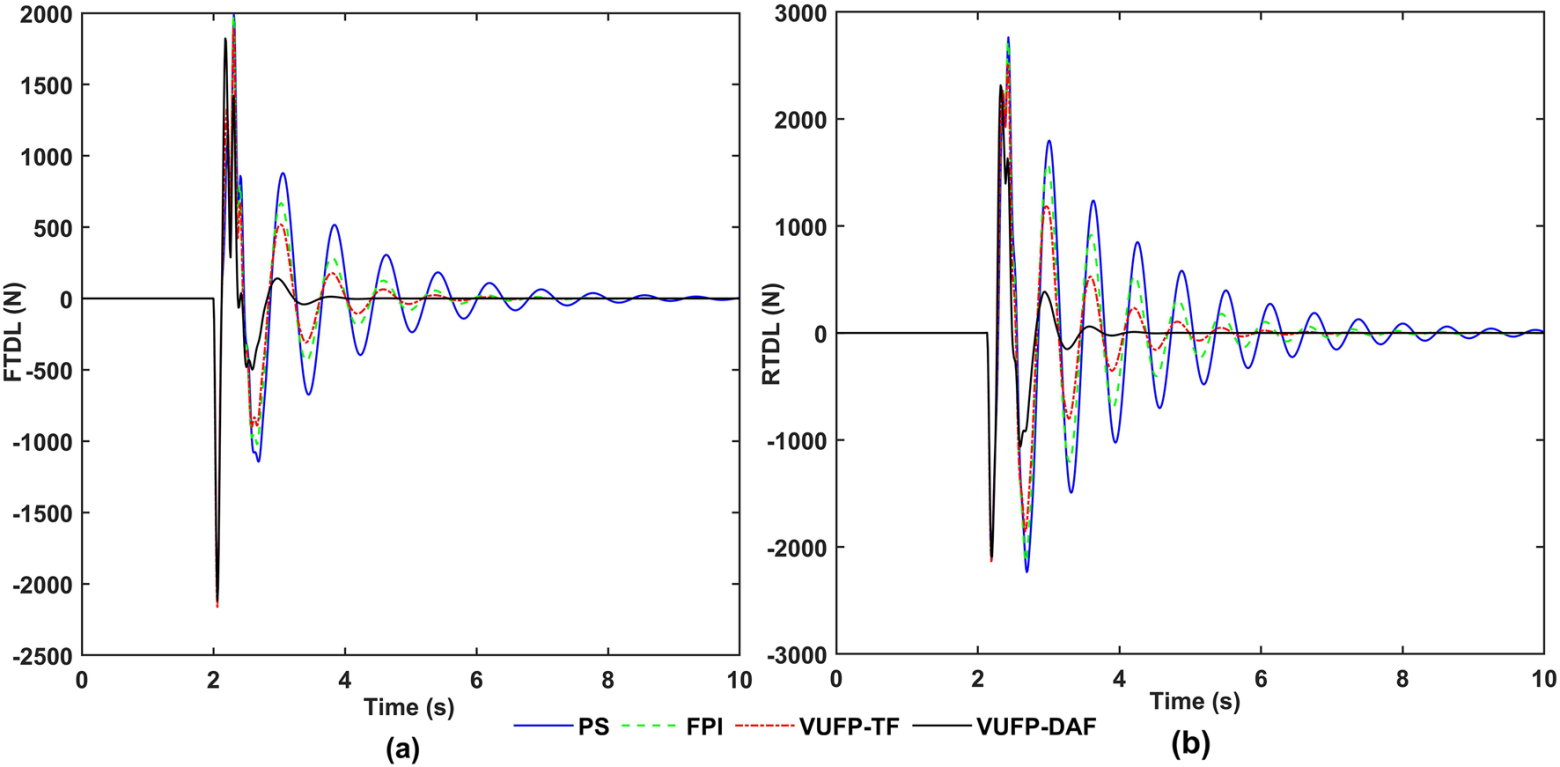
The time-domain response curves of (**a**) FTDL, (**b**) RTDL.

The time-domain response curves of VBA, PAA, FSD, RSD, FTDL and RTDL for PS, FPI, VUFP-TF and VUFP-DAF were calculated when the vehicle was driven on the bump road at a speed of 20 m/s as shown in Figs. 11, 12 and 13, respectively. The RMS values of the corresponding indicators are shown in Table 4.

**Table 4:** Performance indexes of suspension systems.

Indexes	PS	FPI	VUFP-TF	VUFP-DAF
VBA (m/s^2^)	0.7832	0.6953(↓11.22%)	0.5991(↓23.51%)	0.3901(↓50.19%)
PAA (rad/s^2^)	0.4587	0.4127(↓10.03%)	0.3687(↓19.62%)	0.3224(↓29.71%)
FSD (m)	0.0084	0.0072(↓14.29%)	0.0067(↓20.21%)	0.0052(↓38.10%)
RSD (m)	0.0092	0.0081(↓11.96%)	0.0069(↓25%)	0.0050(↓45.65%)
FTDL (N)	346.2	302.8(↓12.54%)	279.9(↓19.15%)	237.6(↓31.37%)
RTDL (N)	585.7	517.4(↓11.66%)	438(↓25.22%)	316.2(↓46.01%)

As can be seen in Fig. 11 and Table 4, compared with PS, the RMS values of VBA and PAA controlled by VUFP-DAF are reduced by 50.19% and 29.71%, respectively. The maximum values of VBA and PAA controlled by VUFP-DAF are also significantly reduced, which can quickly suppress the vehicle vibration. Compared with PS, FPI and VUFP-TF, VUFP-DAF has lower FSD, RSD, FTDL and RTDL indexes, shorter stabilization time and better control effect in Figs. 12 and 13. And the RMS values of FSD, RSD, FTDL and RTDL controlled by VUFP-DAF are reduced by 38.10%, 45.65%, 31.37% and 46.01%, respectively, which can effectively suppress the vehicle body vibration and improve the ride comfort of vehicles. Therefore, VUFP-DAF controller is also applicable to bump road, and has strong robustness and adaptability.

In summary, the references^15,16,28,31,33,35,37^ all used the specific functions to design the scaling factor controller. The references^11,17,18,32^ all used the fuzzy reasoning to design the scaling factor controller. We take references^32,33^ as examples to compare and analyze their advantages and disadvantages with the proposed novel scaling factor controller in this paper. The analysis results are shown in Table 5.

**Table 5:** Analysis results of different scaling factors.

Reference	The type of scaling factors	Advantages and disadvantages
^32^	Fuzzy reasoning	The determination of fuzzy rules of the fuzzy scaling factor overly relies on expert experience, unable to obtain complete fuzzy control rules and not applicable to actual engineering structures
^33^	Specific function	The parameters $\tau_{i}$ of the functional scaling factor are artificially selected constants based on expert experience or similar systems and cannot applicable to any system
The paper	Dynamic adjustment function	Established a novel scaling factor controller. The parameter $\tau_{i}$ of the novel scaling factor can be adjusted in real-time based on the system error *e*(*t*) and its change rate *ec*(*t*), is more flexible and practical in real applications. The proposed VUFP-DAF control strategy has strong practicability and good control performance

## Conclusions

In order to solve the problems that the fuzzy rules rely excessively on expert experience and the contracting-expanding factor with fixed parameters cannot achieve the domain adaptivity, VUFP-DAF algorithm for vehicle semi-active suspension system is proposed. According to the established 1/2 vehicle semi-active suspension dynamics model, the effectiveness of VUFP-DAF is verified by combining the simulation experiments under different working conditions, and the following conclusions are obtained.

1. A novel scaling factor controller is designed according to *e*(*t*) and *ec*(*t*) to achieve the domain adaptivity, and the stability of the proposed scaling factor is proved from five aspects such as duality, non-zero, monotonicity, regularity, and coordination.
2. The proposed VUFP-DAF controller has better control effects at different working conditions. Compared with PS, the VBA and PAA can be reduced by about 45% and 25%, and the FSD, RSD, FTDL and RTDL can be reduced by about 40%, 45%, 30% and 40%, respectively. However, the VBA, PAA, FSD, RSD, FTDL and RTDL controlled by FPI can be reduced by about 10%, 5%, 10%, 10%, 1% and 1%, respectively. And the VBA, PAA, FSD, RSD, FTDL and RTDL controlled by VUFP-TF can be reduced by about 20%, 15%, 25%, 25%, 10% and 10%, respectively. And the VUFP-DAF control effect is significantly better than VUFP-TF and FPI.
3. Simulation results under different operating conditions show that VUFP-DAF controller has strong adaptivity to operating conditions and is very suitable for vibration control of vehicle suspension systems with variable operating conditions.

## Data Availability

The data used to support this research are included within this paper.
